# Brain motion networks predict head motion during rest- and task-fMRI

**DOI:** 10.3389/fnins.2023.1096232

**Published:** 2023-04-11

**Authors:** Dardo Tomasi, Nora D. Volkow

**Affiliations:** ^1^National Institute on Alcohol Abuse and Alcoholism, Bethesda, MD, United States; ^2^National Institute on Drug Abuse, Bethesda, MD, United States

**Keywords:** head motion, fMRI, proprioception, self-motion perception, hyperactivity, impulsivity, machine learning

## Abstract

**Introduction:**

The capacity to stay still during scanning, which is necessary to avoid motion confounds while imaging, varies markedly between people.

**Methods:**

Here we investigated the effect of head motion on functional connectivity using connectome-based predictive modeling (CPM) and publicly available brain functional magnetic resonance imaging (fMRI) data from 414 individuals with low frame-to-frame motion (Δ*d* < 0.18 mm). Leave-one-out was used for internal cross-validation of head motion prediction in 207 participants, and twofold cross-validation was used in an independent sample (*n* = 207).

**Results and Discussion:**

Parametric testing, as well as CPM-based permutations for null hypothesis testing, revealed strong linear associations between observed and predicted values of head motion. Motion prediction accuracy was higher for task- than for rest-fMRI, and for absolute head motion (*d*) than for Δ*d*. Denoising attenuated the predictability of head motion, but stricter framewise displacement threshold (FD = 0.2 mm) for motion censoring did not alter the accuracy of the predictions obtained with lenient censoring (FD = 0.5 mm). For rest-fMRI, prediction accuracy was lower for individuals with low motion (mean Δ*d* < 0.02 mm; *n* = 200) than for those with moderate motion (Δ*d* < 0.04 mm; *n* = 414). The cerebellum and default-mode network (DMN) regions that forecasted individual differences in *d* and Δ*d* during six different tasks- and two rest-fMRI sessions were consistently prone to the deleterious effect of head motion. However, these findings generalized to a novel group of 1,422 individuals but not to simulated datasets without neurobiological contributions, suggesting that cerebellar and DMN connectivity could partially reflect functional signals pertaining to inhibitory motor control during fMRI.

## Introduction

Head motion causes artifacts during magnetic resonance imaging (MRI; Friston et al., 1996; Rohde et al., 2004) and other neuroimaging modalities (Cooper et al., 1992; Nehmeh and Erdi, 2008; Catana et al., 2011), which is why patients are instructed not to move their heads during scanning. However, the capacity to lie still during scanning varies markedly between people, being dependent among other factors on brain maturation and hence much worse in children than adults (Poldrack et al., 2002). It is also impaired in some neurodevelopmental and neurodegenerative disorders such as attention deficit hyperactivity disorder (ADHD), autism, and dementias (Maknojia et al., 2019).

Head motion is particularly concerning for MRI studies of brain functional connectivity (Birn et al., 2006; Power et al., 2012; Satterthwaite et al., 2012; Van Dijk et al., 2012) because it can cause systematic group differences in connectivity (Andrews-Hanna et al., 2007; Fair et al., 2007; Damoiseaux et al., 2008; Fair et al., 2008; Greicius, 2008; Dosenbach et al., 2010) and can even mimic trait correlates of behavior (Siegel et al., 2017). While functional MRI (fMRI) studies treat motion-related signals as artifacts, removing imaging data with excessive motion (Power et al., 2012; Satterthwaite et al., 2012; Van Dijk et al., 2012; Fassbender et al., 2017; Hong et al., 2019; Maknojia et al., 2019), most studies have not investigated if group differences in head movement explain the reported connectivity differences between patients and controls (Buckner et al., 2013). Also, the time frames that drive whole-brain functional connectivity are almost never among those censored for excessive in-scanner motion (Betzel et al., 2022).

Nonetheless the previously reported association between default mode network (DMN) connectivity and in-scanner head motion during resting-state fMRI could reflect greater self-referential mental activity, which could facilitate the person’s ability to stay still during scanning. Specifically, higher functional connectivity between distant default-mode network (DMN) regions was reported in subjects with low head motion compared to those with high motion (Zeng et al., 2014). However, this study did not find within-subject differences in connectivity between fMRI sessions with low and high motion (Zeng et al., 2014), suggesting that between subjects differences in head motion reflect a neurobiological trait (Pujol et al., 2014). However, no study to our knowledge has evaluated whether functional connectivity can predict an individual’s head motion behavior, nor what regions or networks predominantly contribute to in-scanner head movement or might be more sensitive to motion artifacts.

Here we tested the hypothesis that individual differences in functional connectivity can be used to predict in-scanner head motion. For this purpose, we analyzed brain imaging data of 414 healthy adults who underwent six different task-fMRI and two rest-fMRI sessions from the Human Connectome Project (HCP). We investigated the reproducibility of head motion prediction in two independent HCP samples, each with 207 healthy individuals, and two novel groups from the Brain Genomics Superstruct Project (GSP), each with 711 healthy young adults.

## Materials and methods

### HCP datasets

The datasets used in this study were extracted from the HCP 1,200 Subjects data release.^1^ HCP participants provided written informed consent as approved by the Institutional Review Board (IRB) at Washington University. To avoid phase encoding bias, the analyses were restricted to participants for whom both phase-encoding scans (left–right, LR; right–left, RL) for the two rest-fMRI sessions (R1 and R2; collected on two different days) and all six task-fMRI sessions (emotion, relational, motor, working memory, language, and gambling; Barch et al., 2013) were complete and available. Individuals were excluded from the study due to incomplete image datasets, image artifacts (identified with the aid of principal component analysis), or excessive head motion (frame-to-frame displacement, Δ*d* > 0.18 mm). We chose this motion threshold to ensure sufficient sensitivity to head motion taking into account that the magnitude of motion-related fMRI signal changes scales with the magnitude of head motion (Satterthwaite et al., 2013b) and that micromotion > 0.2 mm can systematically bias estimates of resting-state functional connectivity (Van Dijk et al., 2012). The 414 participants were half-split into the Training sample for the optimization of prediction models, and the Test sample for the twofold cross-validation of the prediction models in an independent set of subjects. The samples were matched so there was no significant age or sex differences between the Training and Test samples (Table 1). Only one family member was kept in the study.

**Table 1:** Demographics and residual time-averaged root-mean-square (RMS) estimates of absolute (*d*) and relative (Δ*d*) motion for the first 150 frames that survived scrubbing with framewise displacement threshold of 0.2 mm for Training and Test HCP samples.

HCP	Training		Test		P		# Frames removed
Age [years]	29(4)		29(4)		ns		
Sex (M/F)	84/123		87/120		ns		
	**d [mm] mean (SD)**	**Δd [mm] mean (SD)**	**d [mm] mean (SD)**	**Δd [mm] mean (SD)**	**(d)**	**(Δd)**	**mean (SD)**
WM	0.14(0.12)	0.029(0.014)	0.12(0.08)	0.028(0.011)	0.03	ns	1.1(3.3)
LAN	0.16(0.14)	0.038(0.017)	0.14(0.15)	0.035(0.014)	ns	ns	0.9(2.4)
REL	0.23(0.16)	0.052(0.024)	0.21(0.11)	0.048(0.019)	ns	ns	1.8(2.8)
MOT	0.19(0.10)	0.043(0.018)	0.17(0.10)	0.041(0.016)	ns	ns	1.8(3.9)
GAM	0.17(0.09)	0.046(0.021)	0.16(0.08)	0.042(0.016)	ns	ns	0.7(1.8)
EMO	0.30(0.22)	0.065(0.030)	0.24(0.11)	0.060(0.024)	0.001	ns	0.7(1.8)
R1	0.04(0.02)	0.010(0.004)	0.04(0.02)	0.009(0.003)	0.05	ns	0.6(1.7)
R2	0.04(0.02)	0.010(0.005)	0.04(0.02)	0.009(0.004)	ns	ns	0.7(1.6)

EMO, emotion; LAN, language; REL, relational; GAM, gambling; MOT, motor; and WM, working memory; R1, rest1; and R2, rest2. #Frames: Average number of frames removed by scrubbing in 0 < *t* < 2 min.

### GSP datasets

In addition, we used imaging data from 1,422 healthy young adults (21.5 ± 2.9 years old; 800 females) from the Brain Genomics Superstruct Project^2^ to cross-validate the prediction in an independent dataset. GSP individuals provided written informed consent approved by the Partners Health Care IRB and the Harvard University Committee on the Use of Human Subjects in Research and agreed to data sharing.

### fMRI tasks

We aimed to test linear associations of head motion with functional connectivity strength during the resting state, and during the performance of cognitive, emotional, and motor fMRI tasks. Thus, in the HCP dataset we selected eight fMRI sessions, including those collected during the resting state (R1 and R2) and during the performance of 6 different tasks, which are described in detail elsewhere and target the following domains (Barch et al., 2013): *Emotion*, EMO (Hariri et al., 2002); *Relational processing*, REL (Smith et al., 2007); *Motor*, MOT (Buckner et al., 2011; Yeo et al., 2011); *N-back working memory*, WM (Barch et al., 2013); *Language*, LAN (Binder et al., 2011); and *Gambling*, GAM (Delgado et al., 2000).

### MRI acquisition and image analyses

#### HCP datasets

Functional images with high spatiotemporal resolution were acquired in a 3.0 T Siemens Skyra scanner (Siemens Healthcare, Erlangen, Germany) with a 32-channel coil using a gradient echo-planar imaging (EPI) sequence (multiband factor 8, repetition time, TR = 720 ms, echo time, TE = 33.1 ms, flip angle 52°, 104 × 90 matrix size, 72 slices, and 2 mm isotropic voxels) with whole brain coverage (including the cerebellum) and automated alignment of slice positioning (e.g., “AutoAlign” mode; Smith et al., 2013; Uğurbil et al., 2013). Scans were repeated twice using left–right (LR) and right–left (RL) phase encoding directions. For the resting-state scans, the scanner room was darkened, and subjects were asked to lie with eyes open and not to fall asleep while fixating on a white cross (on a dark background) think of nothing, relax, and to remain still during scanning. The T1-weighted 3D magnetization-prepared gradient-echo image (Mugler and Brookeman, 1990; MP-RAGE; TR/TE = 2,400/2.14 ms, TI = 1 s, FA = 8°) and variable flip angle turbo spin-echo (Mugler et al., 2000; Siemens SPACE; TR/TE = 3,200/565 ms) pulse sequences were used to acquire high-resolution anatomical brain images with 0.7 mm isotropic voxels and field-of-view (FOV) = 224 mm × 224 mm. We used the “minimal preprocessing” datasets released by the HCP, which include gradient distortion correction, rigid-body realignment, field-map processing, and spatial normalization to the stereotactic space of the Montreal Neurological Institute (MNI; Glasser et al., 2013).

#### GSP datasets

Imaging data were collected on matched 3 T Tim Trio scanners (Siemens Healthcare, Erlangen, Germany) at Harvard University and Massachusetts General Hospital using a 12-channel phased-array head coil. Gradient-echo EPI (TR = 3 s; TE = 30 ms; flip angle = 85°, 47 slices, 3 mm isotropic resolution; 124 measurements) with whole-brain coverage, including the entire cerebellum, was used to acquire functional images with blood oxygenation level-dependent (BOLD) contrast. Participants were instructed to remain still, stay awake, and keep their eyes open during fMRI. Multi-echo T1-weighted magnetization-prepared gradient-echo (MP-RAGE; van der Kouwe et al., 2008) imaging (TR = 2.2 s; TE = 1.5/3.4/5.2/7.0 ms; flip angle = 7°; TI = 1.1 s; 144 slices, 1.2 mm isotropic resolution) was used to acquire anatomical images. The FreeSurfer (version 5.3.0) package^3^ (Fischl et al., 2002) was used to automatically segment anatomical MRI scans into cortical and subcortical gray matter structures. Functional images were screened for artifacts and excessive motion. The first four image volumes were discarded for signal stabilization purposes. The University of Oxford’s Center for Functional Magnetic Resonance Imaging of the Brain (FMRIB) Software Library (FSL version 5.0)^4^ was used for image realignment (to correct for head motion with MCFLIRT, Motion Correction using FMRIB’s Linear Image Registration Tool), and for spatial normalization to the MNI152 template using 3 mm isotropic voxels (with FLIRT, the FMRIB’s Linear Image Registration Tool; Jenkinson et al., 2002; Smith et al., 2004).

In addition, displacement timeseries reflecting how much a given voxel moved as a function of time were simulated by applying the affine transformations from image realignment to the first volume (Satterthwaite et al., 2013a). These simulated time series were realigned and spatially normalized to the MNI space.

#### Head motion

The Euclidian norms of head displacement and frame-to-frame velocity, $d_{i}$ and $\Delta d_{i}$ , were calculated from image realignment parameters (translations along *x*, *y*, and *z* with respect to the first volume) for each timepoint, *i*:

(1) $$d_{i}=\sqrt{x_{i}^{2}+y_{i}^{2}+z_{i}^{2}};\Delta d_{i}=\sqrt{{\left(x_{i}-x_{i-1}\right)}^{2}+{\left(y_{i}-y_{i-1}\right)}^{2}+{\left(z_{i}-z_{i-1}\right)}^{2}}$$

and the average root-mean-square (RMS) values of $d_{i}$ and $\Delta d_{i}$ , across LR and RL scans and timepoints, were used as summary metrics of absolute (*d*; measured from t = 0) and relative (frame-to-frame; Δ*d*) head motion, respectively, in mm.

Framewise displacements (FD) were computed for every time point from head translations and rotations:

[2] $$\begin{array}{l} FD_{i}=\left|x_{i}-x_{i-1}\right|+\left|y_{i}-y_{i-1}\right|+\left|z_{i}-z_{i-1}\right|+50mm\times \\ \left(\left|\alpha_{i}-\alpha_{i-1}\right|+\left|\beta_{i}-\beta_{i-1}\right|+\left|\gamma_{i}-\gamma_{i-1}\right|\right) \end{array}$$

where rotational angles, α, β, and γ (in radians) were converted to displacements on the surface of a sphere of radius 50 mm as in previous work (Power et al., 2012). Time points were excluded if the RMS change in BOLD signals frame-to-frame was larger than 0.5%, and exceeded the censoring threshold FD_i_ > 0.5 mm (or FD_i_ > 0.2 mm; Power et al., 2015). Global signal regression (GSR) was used in all evaluated data to remove non-neuronal sources that contribute to the global signal (Power et al., 2014). Low-pass filtering (0.10 Hz frequency cutoff) was used to attenuate physiologic noise of high-frequency components.

#### Effect of pipeline choices for noise suppression

To assess the effect of motion on functional connectivity we studied fMRI datasets with and without removal of motion-related signals using linear regression with the time-varying realignment parameters (Tomasi and Volkow, 2010) and independent component analysis (ICA)-based X-noiseifier, an ICA-based automatic noise detection algorithm that can minimize various types of noise sources including head motion (Salimi-Khorshidi et al., 2014).

#### Functional connectome

Connectivity matrices, **M**, were constructed for each fMRI dataset and subject, using the corresponding preprocessed 4D time series. To assess the functional connectivity between regions-of-interest (ROIs) we used the Interactive Data Language (IDL, L3Harris Geospatial, Broomfield, CO). Three different brain atlases were used to provide ROIs: 1) Automated Anatomical Labeling (AAL)—Tzourio-Mazoyer et al. (2002) and 2) Shen et al. (2013), both of which include the cerebellum and subcortical regions and 3) Gordon et al. (2016), which does not include the cerebellum and subcortical regions, to assess the effect of brain parcellation on the accuracy of the behavioral prediction model. In addition, we combined the cortical partitions of the Gordon atlas with the 26 subcortical (including the brainstem) and 41 cerebellar partitions of the Shen atlas in a new whole-brain atlas with 400 partitions (Gordon400). Pearson correlation coefficients between pairs of ROI time courses were calculated independently for LR and RL scans and normalized to z-scores using the Fisher transformation. This resulted in 116 × 116 (AAL), 268 × 268 (Shen), 333 × 333 (Gordon), and 400 × 400 (Gordon400) symmetric connectivity matrices for each fMRI session and participant. The LR and RL correlation matrices corresponding to the same functional session were averaged to increase signal-to-noise. To allow for comparisons between task- and rest-fMRI results that were not biased by unequal data sampling, only the first 150 frames (to correspond with the short duration of the Emotion task-fMRI; ~2 min; 176 time points) of the time series that survived scrubbing were used to compute the corresponding **M**.

### Head motion prediction model

The optimization of the prediction models was carried out using connectome-based predictive modeling (CPM; Shen et al., 2017) using leave-one-out cross-validation. Specifically, at each of *n* iterations, one of the *n* individuals was excluded and the four CPM steps, *feature selection*, *feature summarization*, *model building*, and *assessment of prediction significance* were carried sequentially *n* times in an iterative fashion as follows. *Feature selection*: Pearson correlation was used to assess associations between head motion scores and each element of the connectivity matrices (M*_ij_*) in the Training sample. Matrix elements that had significant positive or negative correlations with the observed head motion scores (RMS values of *d* and Δ*d*) were identified as edges of the positive or negative adjacency matrices and included in the model. Two thresholds were tested (*p* < 0.01 or 0.05) for feature selection to ascertain that results did not depend on arbitrary threshold selection. *Feature summarization*: Edges with positive (negative) correlation with motion scores were added to compute the positive (negative) network strength, X (Y). *Model building*: a bilinear model was fitted to the data across the *n*-1 individuals.

(3) Ψ=a+bX+cY

Here *a*, *b*, and *c* are model parameters, Ψ is the observed head motion score, and X and Y are the positive and negative network strengths derived from the connectivity matrices. We also assessed linear models purely driven by positive or negative features by setting *c* = 0 or *b* = 0. *Assessment of prediction significance*: The model was then used to predict the head motion score of the remaining individual from his/her corresponding positive and negative network strengths.

In addition, we used a twofold cross-validation approach to assess how the CPM results generalize to an independent data set. Specifically, the CPM model and features derived from the Training sample were used to predict head motion in the independent Test sample. Finally, the Training and Test samples were swapped (e.g., the CPM model and features derived from the Test sample were used to predict head motion in the independent Training sample) to complete the cross-validation.

### Statistical analyses

The Shapiro–Wilk normality test (Shapiro and Wilk, 1965) was used to confirm the normal distribution of the functional connectivity strength. Thus, Pearson correlation was used to assess prediction accuracy, unless otherwise specified. Since Training and Test were independent samples, we used parametric statistics to assess the statistical significance of group differences in correlation between observed and predicted motion scores. To test for differences between two dependent correlations sharing one variable we used the Williams’s test (Williams, 1959), and for correlations with different variables we used the Steiger’s test (Steiger, 1980). The cortical networks were labeled using the Yale network definitions (Noble and Scheinost, 2020). Statistically significant correlations for a sample size *n* = 207 were set at *p* < 3.213E-03, corresponding to R = 0.204, using Bonferroni corrections for 16 comparisons (8 fMRI sessions × 2 motion measures). The Bonferroni method was also used to correct for multiple comparisons the results from within- and between-network predictions with the Gordon400 parcellation atlas (14 networks). Specifically, Bonferroni corrections were carried with 14 (within-network; R > 0.2) or 91 (between-network; R > 0.23) comparisons. A permutation framework was used for null hypothesis significance testing. Specifically, 1,000 random permutations of head motion scores were used to assess the distribution of prediction accuracy under possible rearrangements of motion scores.

## Results

**Figure 1:**
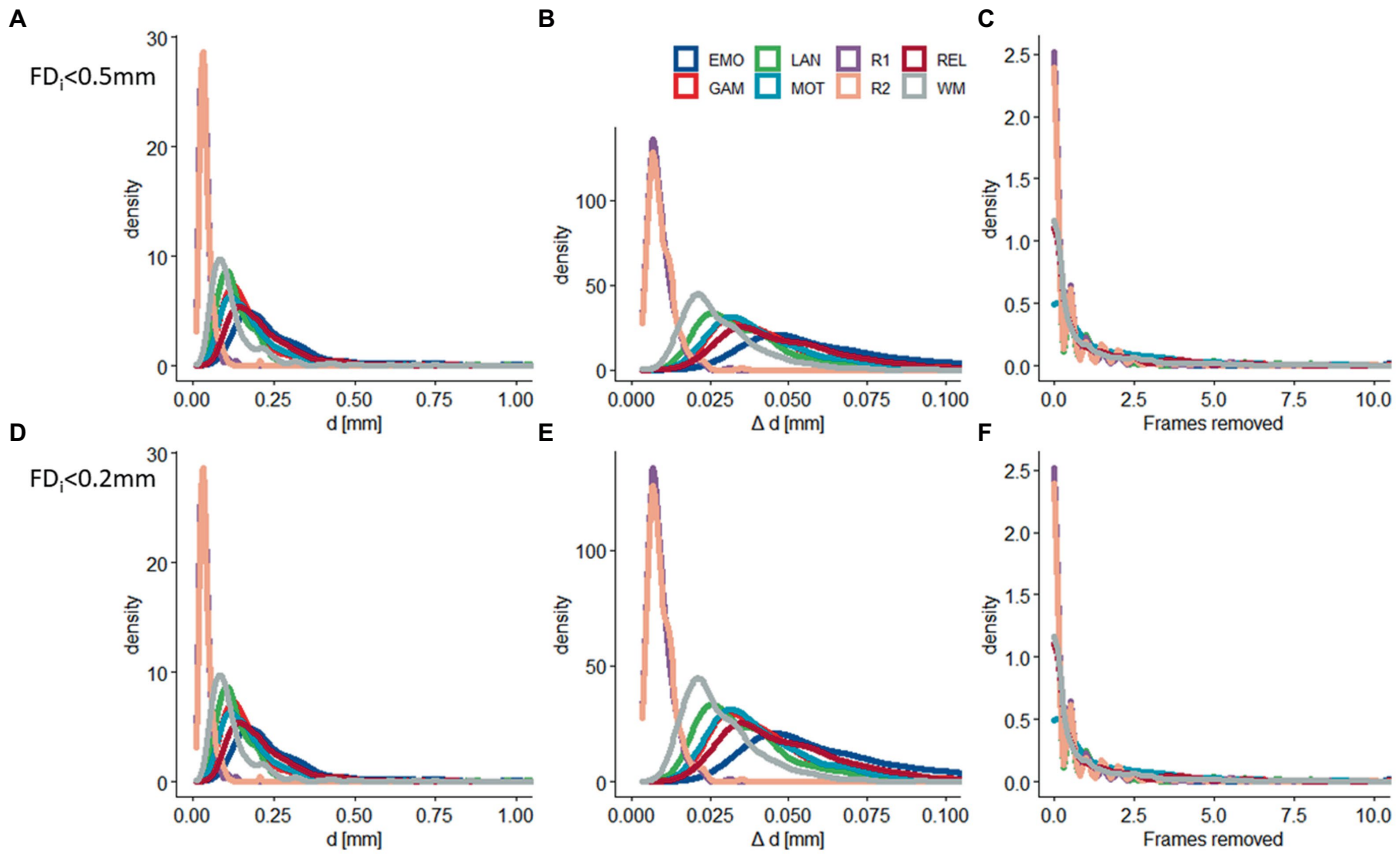
Residual head motion. Density plots showing across-subjects’ distributions of residual time-averaged root-mean-square (RMS) estimates of absolute (*d*) and relative (Δ*d*) motion, and the number of frames removed with framewise displacement (FD) thresholds of 0.5 mm **(A–C)** and 0.2 mm **(D–F)** for 6 task-fMRI sessions: (EMO: emotion; LAN: language; REL: relational; GAM: gambling; MOT: motor; and WM: working memory) and 2 rest-fMRI session (R1: rest1; and R2: rest2). Sample size: 414 healthy young adults.

The realignment estimates of motion (*d*; i.e., “absolute motion”) were significantly lower for rest (*d* = 0.04 mm ± 0.02 mm) than for task sessions (*d* = 0.19 mm ± 0.14 mm; *p* < 2E-16; Figure 1). Similarly, the frame-to-frame motion estimates (Δ*d*; i.e., “relative motion”) were significantly lower for rest (Δ*d* = 0.010 ± 0.004 mm) than for task sessions (Δ*d* = 0.044 mm ± 0.022 mm; Figure 1). There were no sex differences, but older age was associated to lower *d* and Δ*d* (*p* < 0.004, *F* = 11.3, df = 3,302, ANOVA).

### Prediction of head motion from fMRI data

**Figure 2:**
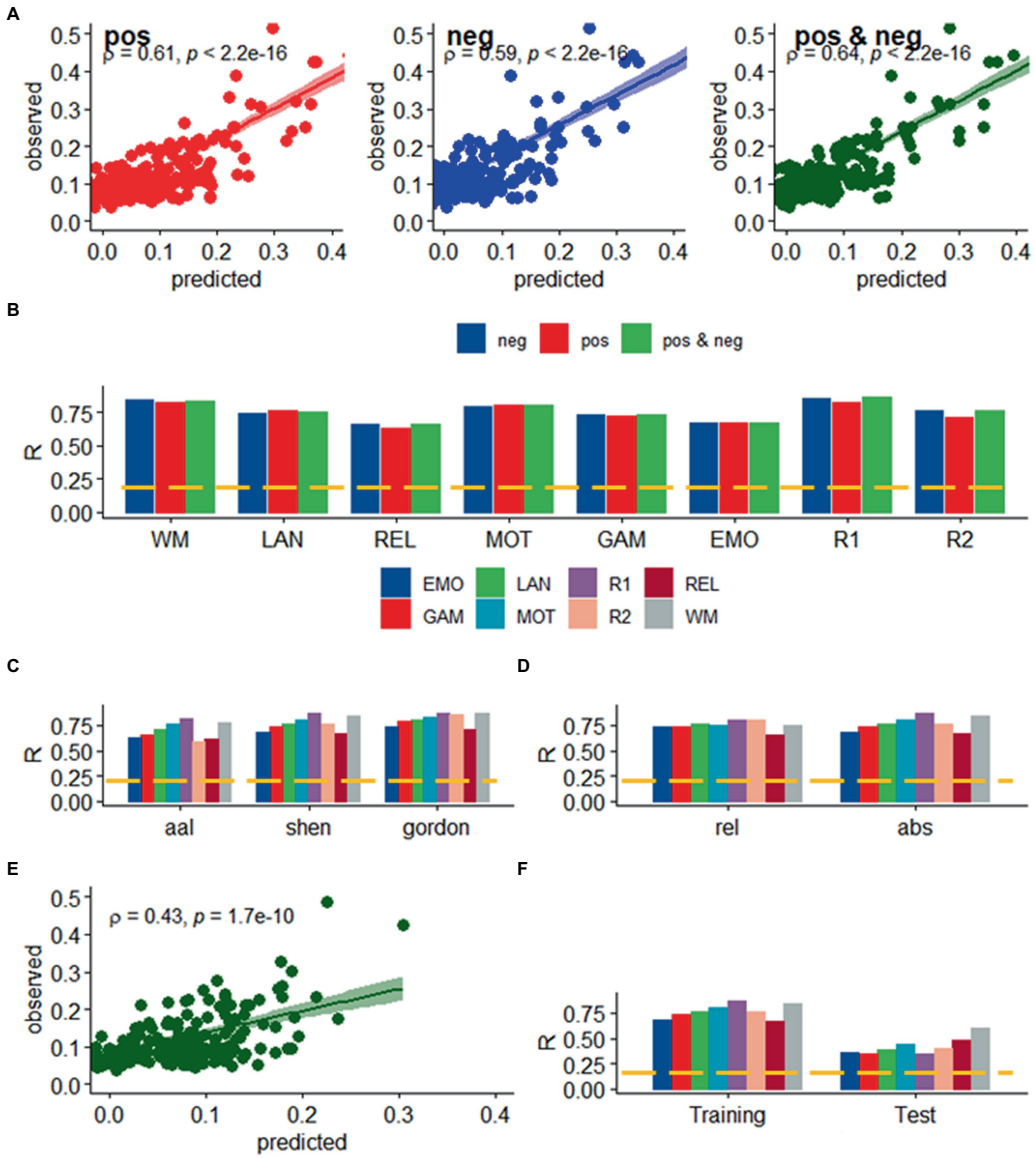
Prediction of absolute head motion. **(A)** Observed absolute head motion during fMRI sessions was predicted from positive and negative network strengths in “left out” individuals of the Training sample (*n* = 207), using leave-one-out cross-validation for working memory (WM). **(B)** Correlation factor (R) between observed and predicted absolute motion excursions (*d*) did not differ across models purely based on positive, negative network strength, or both. Prediction accuracy (*R*) across brain parcellations **(C)** and for absolute and relative motion (Δ*d*; **D**) in the training sample. **(E)** Functional connectivity predicted absolute head motion in an independent set of individuals (“Test sample”; *n* = 207) using optimal models and features derived from the Training sample for the bilinear model for WM. **(F)** Prediction accuracy for absolute motion in Test and Training samples. ---*p* < 0.05, Bonferroni corrected for 16 comparisons. Shen parcellation atlas. EMO, emotion; LAN, language; REL, relational; GAM, gambling; MOT, motor; and WM, working memory; R1, rest1; and R2, rest2. Sample size: 414 healthy young adults. Censoring threshold 0.5 mm.

**Figure 3:**
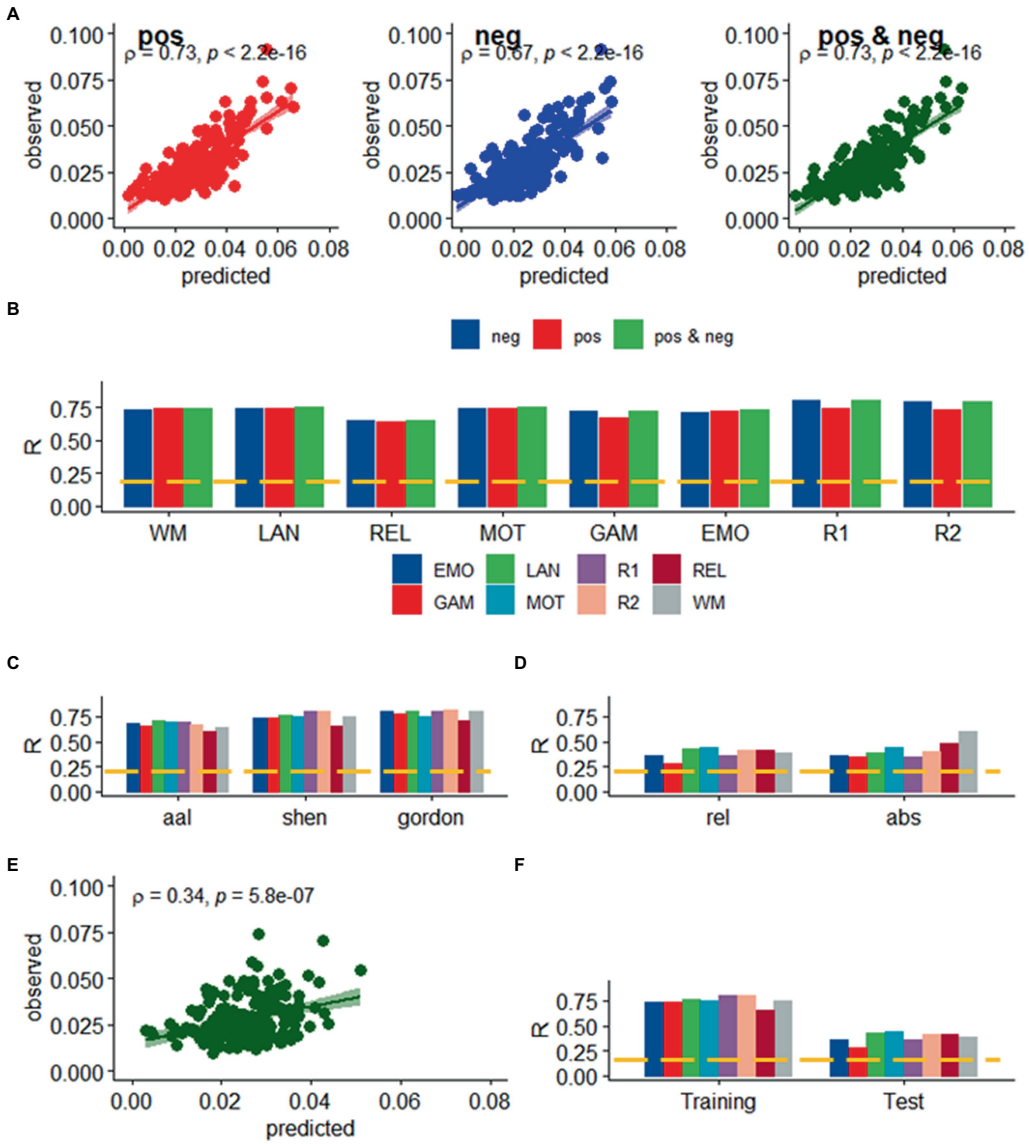
Prediction of relative head motion. **(A)** Observed relative head motion during fMRI sessions was predicted from positive and negative network strengths in “left out” individuals of the Training sample (*n* = 207), using leave-one-out cross-validation. **(B)** Correlation factor (R) between observed and predicted relative motion excursions (Δ*d*), as a function of fMRI session and model. Prediction accuracy (*R*) across brain parcellations in the training sample **(C)** and for absolute and relative motion (Δ*d*; **D**) in the test sample. **(E)** Functional connectivity predicted relative head motion in an independent set of individuals (“Test sample”; *n* = 207) using optimal models and features derived from the Training sample for the bilinear model for WM. **(F)** Prediction accuracy for relative motion in test and training samples. ---*p* < 0.05, Bonferroni corrected for 16 comparisons. Shen parcellation atlas. EMO, emotion; LAN, language; REL, relational; GAM, gambling; MOT, motor; and WM, working memory; R1, rest1; and R2, rest2. Sample size, 414 healthy young adults. Censoring threshold 0.5 mm.

We found a strong linear association between observed and predicted values of *d* using Spearman correlation (ρ > 0.59; *p* < 2.2E-16; Figure 2A). Prediction models based on positive and negative network strength performed similarly across fMRI sessions in the Training sample (Figure 2B), in agreement with prior studies (Finn et al., 2015), supporting the notion that the negative and positive networks contain redundant information (Rosenberg et al., 2016). The correlation between observed and predicted absolute motion scores across subjects (“*R,”* a benchmark of prediction accuracy) did not differ between task- or rest-fMRI sessions across all parcellations (*p* > 0.3, 2-sided *t*-test). Across linear and bilinear models, prediction accuracy was higher for Gordon than Shen and AAL and for Shen than AAL parcellations (*p* < 1E-03, 2-sided paired *t*-test; Figure 2C). The linear associations between observed and predicted measures of Δ*d* were like those of *d* (Figure 3). Across fMRI sessions and models, motion prediction accuracy was lower for Δ*d* than *d*, independently for the Training and Test sessions (*p* < 0.01, 2-sided paired *t*-test, Figures 2D, 3D).

### Validation in an independent sample

We confirmed the generalizability of the linear association between head motion and functional connectivity in the Test sample (e.g., using twofold cross-validation). Specifically, for each participant in the independent Test sample (*n* = 207) we predicted head motion from the positive and negative features and the model parameters derived from the Training sample (Figure 2E). In the Test sample, prediction accuracy was lower for Gordon than Shen and AAL parcellation (*p* < 0.01, 2-sided paired t-test). In the Test sample, we also found that all prediction models performed similarly across fMRI sessions. Δ*d*-prediction accuracy was lower for the Test sample than for the Training sample (*p* < 5E-07, 2-sided paired *t*-test, df = 23; Figure 3F).

### Sensitivity to motion

Functional connectivity studies frequently address motion concerns by minimizing BOLD signals associated with head motion. Popular approaches for this are based on (1) linear regression of rigid-body realignment parameters (Satterthwaite et al., 2013a) and ICA-based denoising algorithms that can minimize various types of noise sources, including head motion (Behzadi et al., 2007; Salimi-Khorshidi et al., 2014; Pruim et al., 2015); and (2) the removal of subjects with excessive micro-motion (Van Dijk et al., 2012). Here we used these approaches to assess the sensitivity of the head motion prediction model to the amount of motion in the data. The removal of 214 individuals from the original cohort of 414 individuals (“moderate motion”) who had micro motion 0.18 mm > Δ*d* > 0.08 mm in at least one of the fMRI sessions resulted in a subsample of 200 individuals with “low motion” (Δ*d* < 0.08 mm). The low-motion subsample was split into 2 groups of 100 individuals for CPM training and testing purposes.

**Figure 4:**
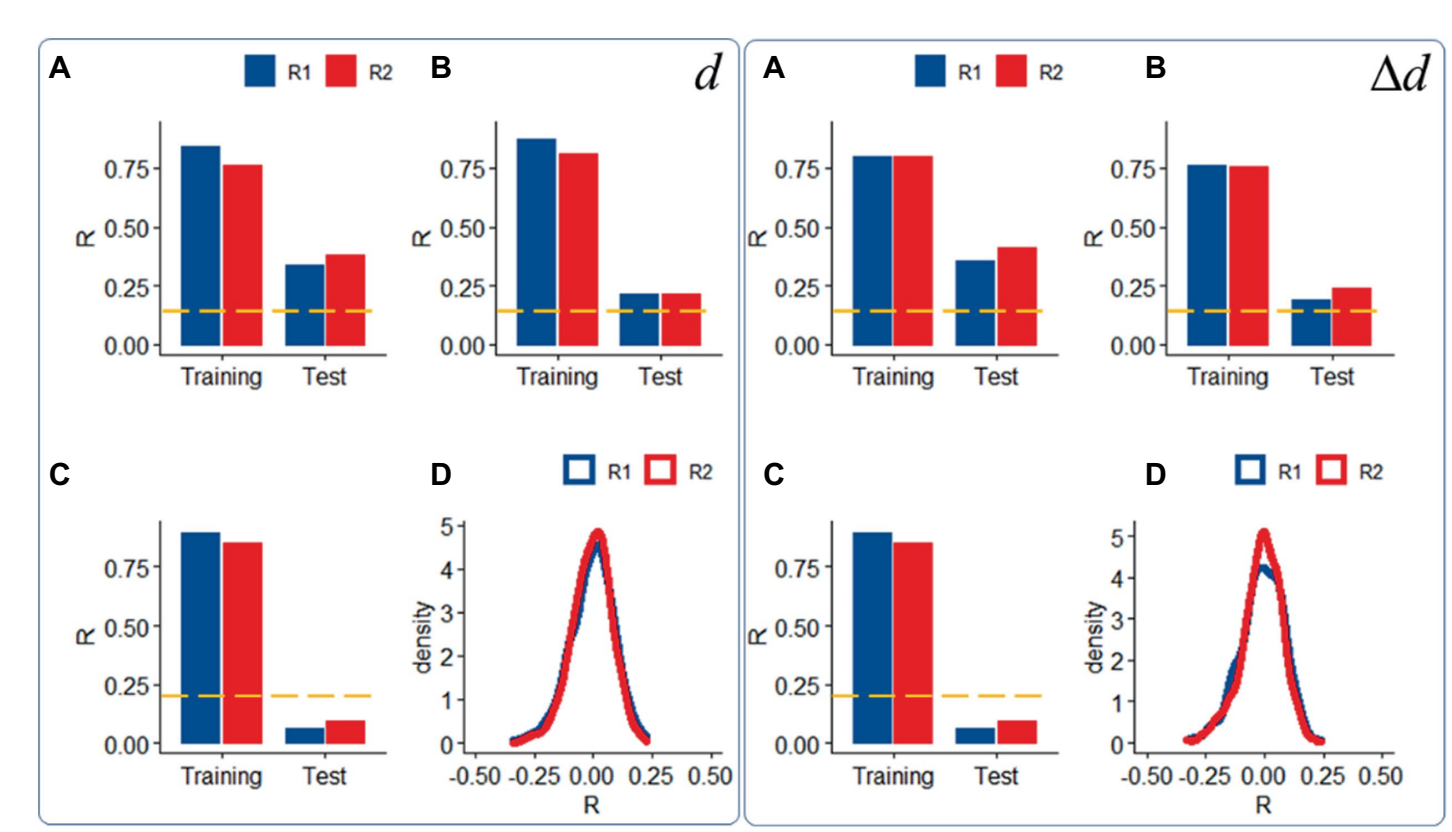
Sensitivity to motion signals: Predictability of relative (right panel) and absolute (left panel) head motion from functional connectivity datasets (R1: rest1; R2: rest2) in the Training (*n* = 207) and Test (*n* = 207) samples with “moderate motion” (Δ*d* < 0.04 mm), without **(A)** and with **(B)** ICA-denoising (i.e., “noise suppression”), and in a “low motion” subsample of 200 (Training: 100; Test = 100) individuals (Δ*d* < 0.02 mm); **(C)** Density plots showing distributions of prediction accuracy obtained from 1,000 random permutations of *d* or Δ*d* across individuals in the moderate motion sample for each fMRI session **(D)**. ---*p* < 0.05, Bonferroni corrected for 2 comparisons. Shen parcellation atlas. Bilinear model. Censoring threshold 0.5 mm.

Compared to datasets with moderate motion, datasets with ICA-based denoising (i.e., “noise suppression”) demonstrated slightly attenuated head motion prediction, independently for *d* and Δ*d* (*p* < 0.05, ANOVA; Figures 4A,B) and those with low motion did not predict *d* or Δ*d* in the Test sample (Figure 4C), consistent with the removal of signals correlated with rigid body motion.

We also confirmed the significance of the findings, against the null hypothesis that functional connectivity would not predict head motion, using a permutation test in which head motion (*d* or Δ*d*) did not correspond to functional connectivity datasets across individuals. Under the null hypothesis, prediction accuracy had a bell-shaped distribution of σ = 0.09 (Figure 4D).

**Figure 5:**
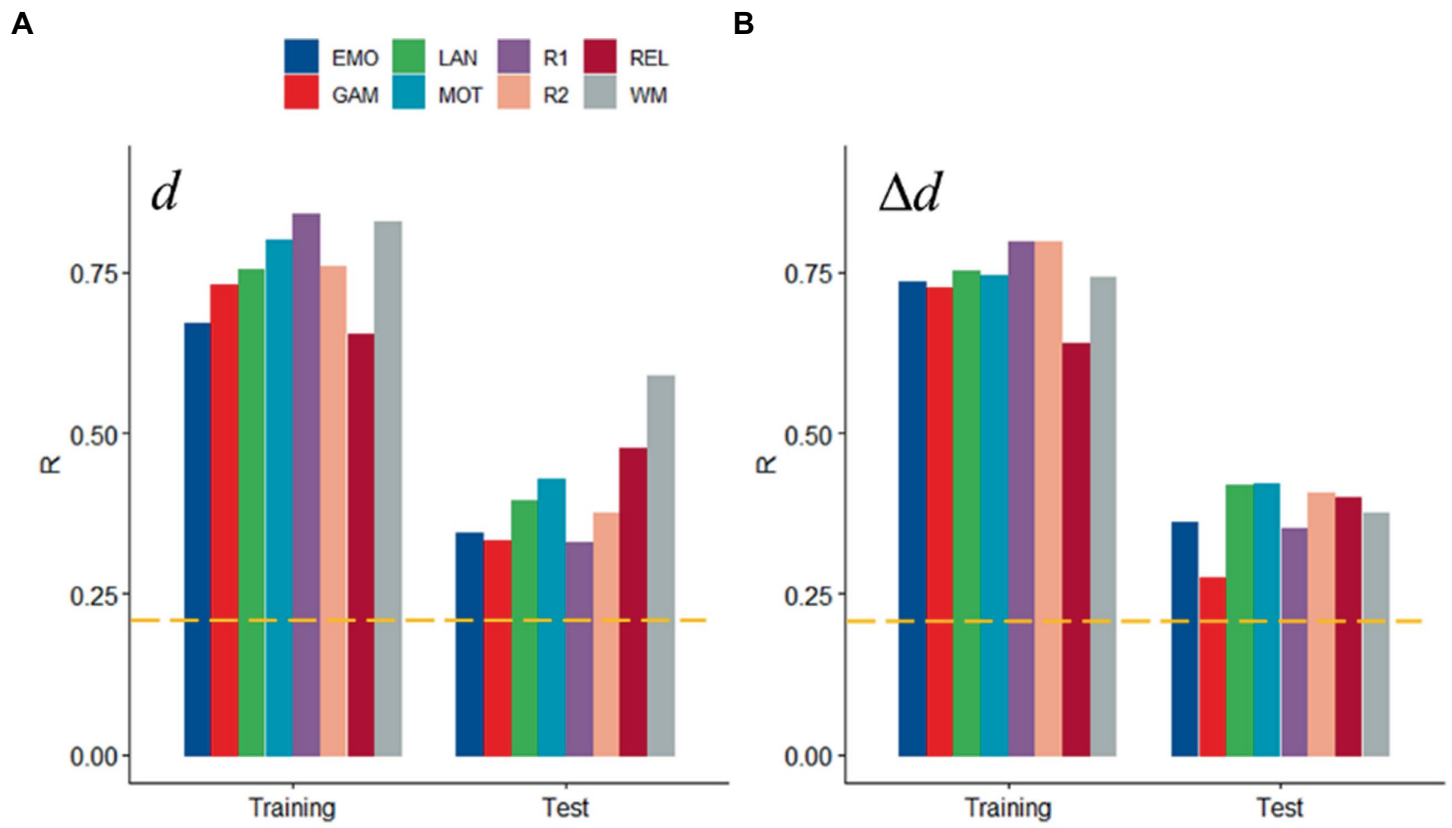
Sensitivity to head motion with censoring threshold 0.2 mm: Absolute (*d*; **A**) and relative (Δ*d*; **B**) motion prediction accuracies from functional connectivity datasets. Training (*n* = 207) and Test (*n* = 207) samples. ---*p* < 0.05, Bonferroni corrected for 16 comparisons. Shen parcellation atlas. Bilinear model.

Scrubbing (censoring) is also a popular approach to control for head motion artifacts in functional connectivity (Power et al., 2012, 2014, 2015). Both for *d* and Δ*d*, prediction accuracy did not differ when computed from datasets with different censoring threshold (FD_i_ < 0.2 mm, Figure 5 vs. FD_i_ < 0.5 mm, Figures 2F, 3F; *p* > 0.15, ANOVA).

### Within- and between-network predictions

**Figure 6:**
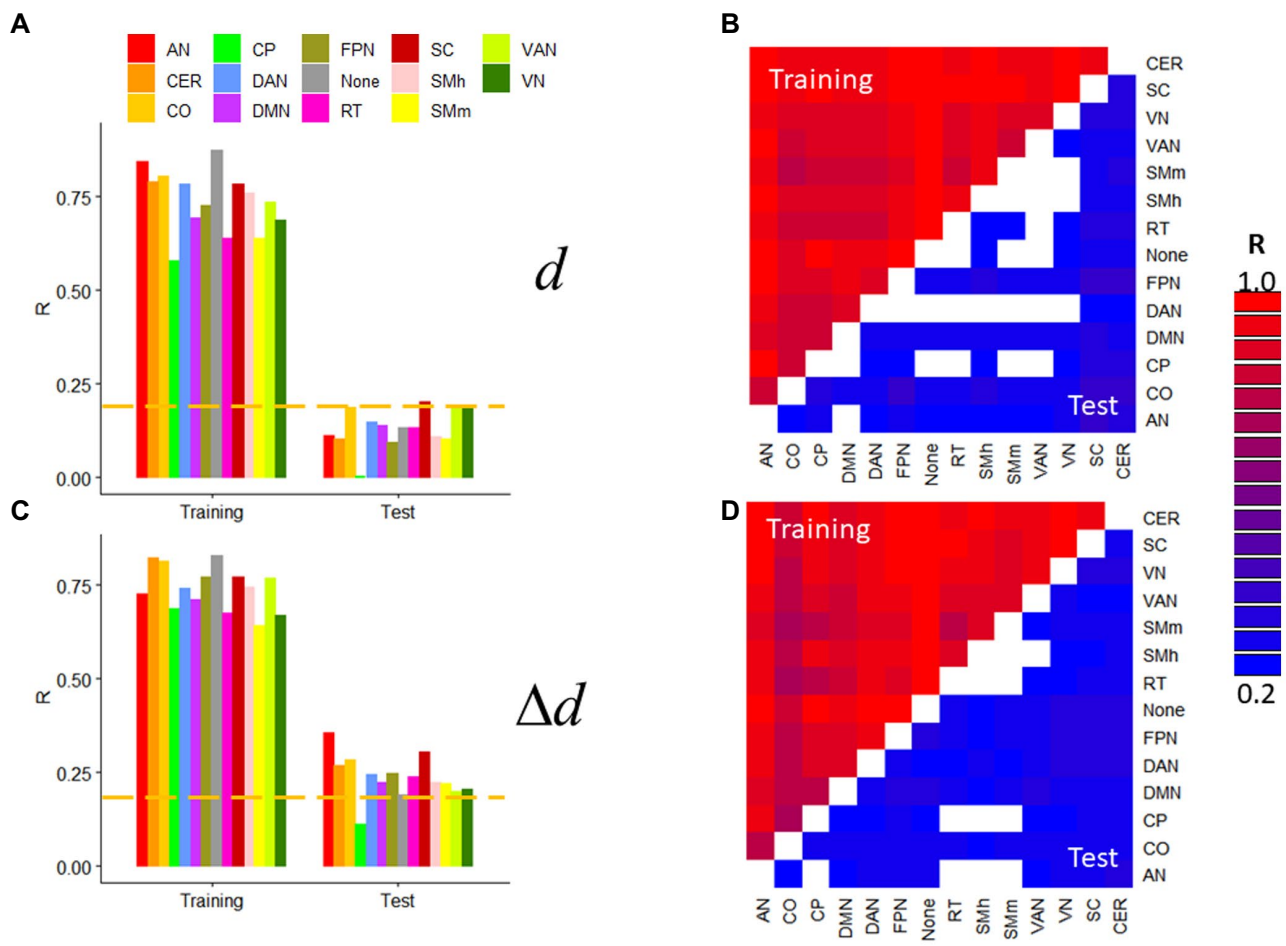
Within- and between-network predictions. Absolute (*d*; **A,B**) and relative (Δ*d*; **C,D**) motion prediction accuracies that emerged from within-network **(A,C)** and between-network (**B,D**; the threshold R > 0.23 corresponds to *p* < 0.05, Bonferroni corrected for 91 comparisons) edges of the functional connectivity matrix from R1. Training (*n* = 207) and Test (*n* = 207) samples. ---*p* < 0.05, Bonferroni corrected for 14 comparisons. Gordon400 parcellation atlas. AN, auditory; CO, cingulum-operculum; CP, cingulum-parietal; DAN, dorsal-attention; DMN, default-mode; FPN, frontoparietal; RT, retrosplenial-temporal; SMh, sensorimotor-hand; SMm, sensorimotor-mouth; VAN, ventral attention; and VN, visual networks; CER, cerebellum; and SC, subcortical regions.

Next, we assessed prediction accuracy for specific network connections by restricting the features to either within-network or between-network edges. We used the 14 resting-state networks in the Gordon400 parcellation and R1 datasets with extremely low motion [*d* = 0.04(0.02) mm; Δ*d* = 0.010(0.004) mm]. *d*- and Δ*d*-prediction accuracies were statistically significant across within- or between-network edges and were higher for Training than Test samples (*p* < 2.2e-16; Figure 6). In the Test sample, prediction accuracy varied significantly across networks when using within-network edges (>54%; Figure 6) but less so when using between-network edges (<27%). In the Test sample, within-network prediction accuracy was significant only for the visual and ventral attention networks, subcortical regions, and the cerebellum but was significant for most between-network edges, independently for *d* and Δ*d* (*p* < 0.05, corrected). Similar results emerged from task-fMRI datasets (not shown).

### Predictions from simulations

**Figure 7:**
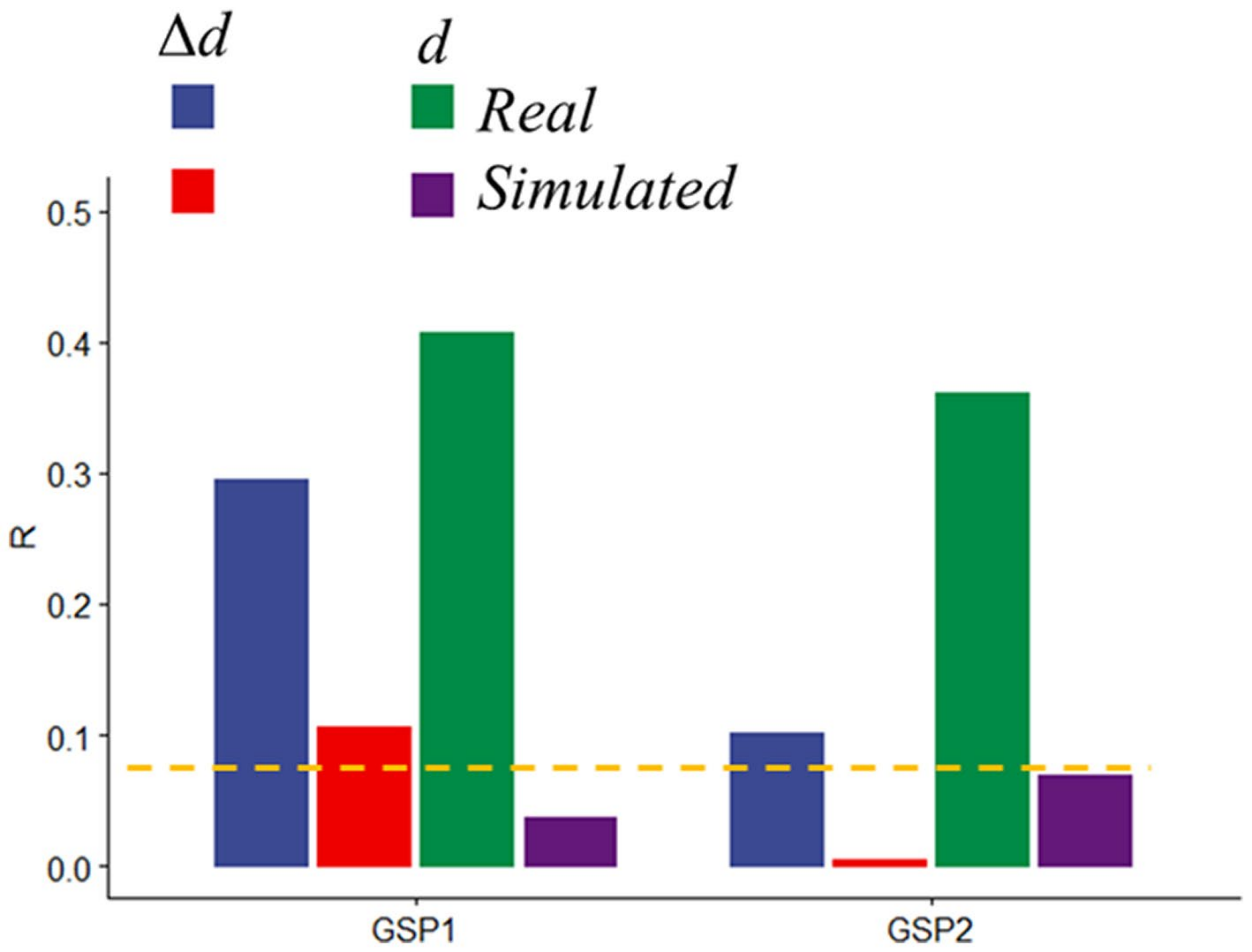
Prediction accuracy in real and simulated data. Absolute (*d*) and relative (Δ*d*) motion prediction accuracies from real and simulated functional connectivity datasets from the Brain Genomics Superstruct Project (GSP1, as the training sample, and GSP2, as the test sample; *n* = 711, each). Brain parcellation: Shen; Model: bilinear. ---*p* < 0.05, Bonferroni corrected.

To rule out potential neuronal contributions to motion prediction we assessed head motion prediction accuracy in simulated datasets in which the time-varying signals reflected only the real translations and rotations of GSP datasets, but not BOLD signal changes. Compared to the significant and reproducible *d*- and Δ*d*-predictions obtained with the real GPS data, prediction accuracy in simulated data was very weak for Δ*d* and did not reach significance for *d*, both for GSP1 and GSP2 (Figure 7).

### Common motion-sensitive network

**Figure 8:**
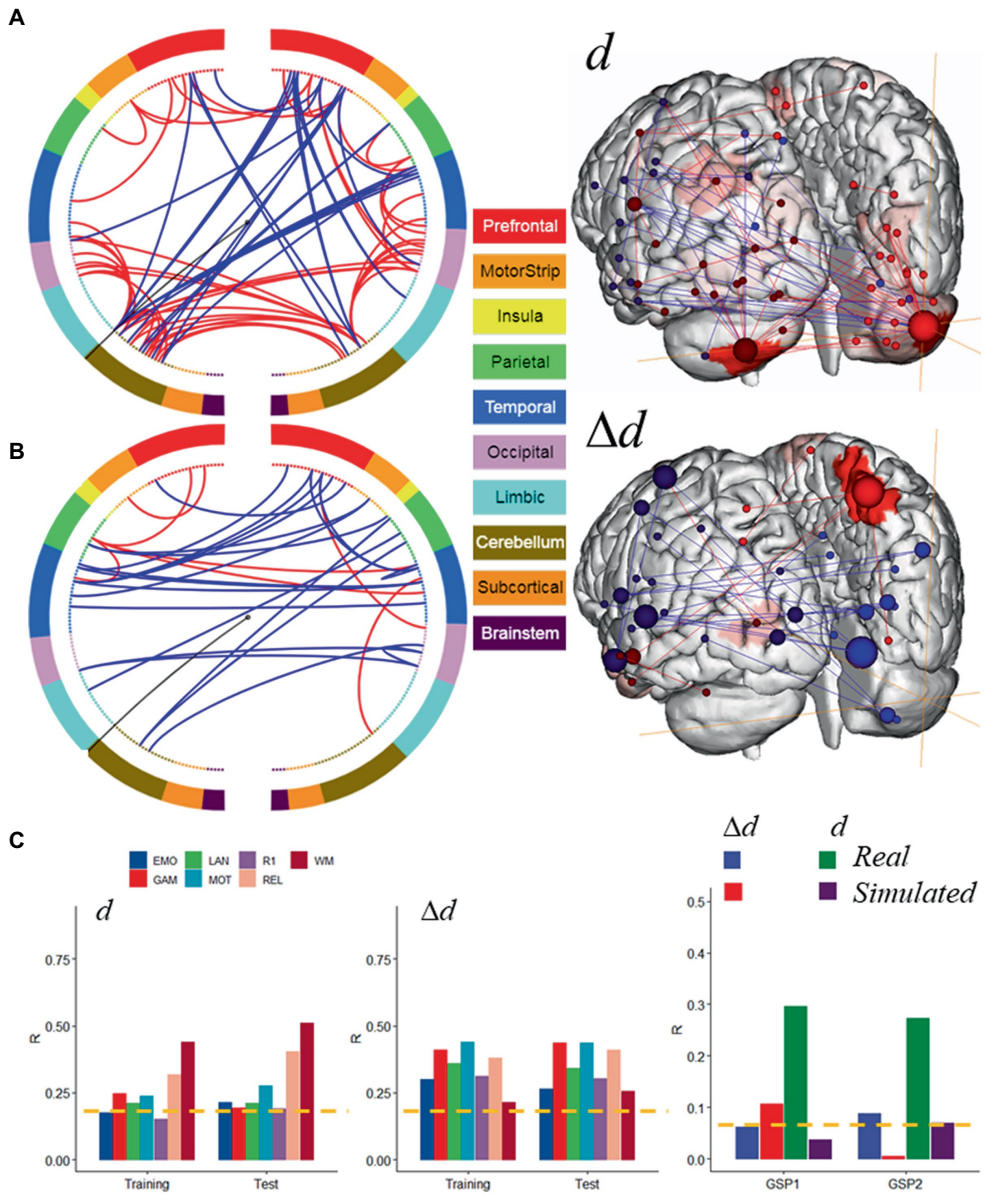
Motion-sensitive networks. Motion-network reflecting the 50% overlap of positive (**─**) or negative (**─**) networks that predicted absolute **(A)** and relative **(B)** head motion across 8 fMRI sessions and Training and Test subsamples, each of 207 individuals with moderate motion, and a glass brain plot where each node is represented as a sphere of size proportional to the number of edges of the node (right). The BioImage Suite Web (https://bioimagesuiteweb.github.io) was used to create these figures. **(C)** The motion-networks predicted absolute and relative head motion for both HCP samples and for real sessions from the Brain Genomics Superstruct Project (GSP1 and GSP2; *n* = 711, each). Brain parcellation: Shen; Model: bilinear. ---*p* < 0.05, Bonferroni corrected. Bilinear model parameters: *a* = 0.016; *b* = 0.005; *c* = −0.010.

Hypothesizing that a unique subset of positive and negative network edges can be used to predict head motion for any of the fMRI sessions and using the moderate motion subsamples we identified edges that overlapped 25% or more across fMRI sessions, independently for positive and negative networks. Using Shen atlas partitions, the overlapping networks that predicted *d* had 100 positive edges and 52 negative edges, which predominantly emanated from bilateral hubs in the cerebellum Crus II (Figure 8A; Table 2) as well as medial DMN (anterior cingulum, superior medial frontal, and inferior temporal cortices) and salience network (SN; insula) regions, and the calcarine cortex (Figure 8A; Table 2). Positive edges predominantly reflected connections to contralateral regions (left–right cerebellum, left–right prefrontal cortex, PFC) and negative edges reflected ipsilateral and contralateral anterior-to-posterior connections (cerebellar-PFC). The overlapping networks that predicted Δ*d* were more restricted than those that predicted *d*, and had 14 positive and 44 negative edges that predominantly emanated from lateral (inferior and middle temporal gyri) and medial (superior medial frontal gyrus and precuneus) DMN, and FPN (left temporal pole) networks (Figure 8B; Table 3).

**Table 2:** Degree and coordinates of the 9 major hubs of the absolute ‘motion-sensitive network’ in the stereotactic space of the Montreal Neurological Institute (MNI).

Node	Region	BA/nucleus	Degree	MNI coord [mm]			Region
				*x*	*y*	*z*	
100	Cerebellum	Crus II	20	32	−78	−40	CB
242	Cerebellum	Crus II	15	−30	−80	−40	CB
155	Insula	47	9	−33	22	6	SN
215	Calcarine	17	5	−6	−81	12	VN
185	Inferior Temporal	21	5	−38	3	−38	DMN
148	Sup medial frontal	8	5	−11	34	51	DMN
140	Ant Cingulum	32	4	−6	48	12	DMN
141	Sup medial frontal	10	4	−12	65	4	DMN

CB, cerebellum. Networks: SN, salience; DMN, default-mode; VN, visual. Node numbers correspond to the Shen atlas.

**Table 3:** Degree and coordinates of the five major hubs of the relative “motion-sensitive network” in the stereotactic space of the Montreal Neurological Institute (MNI).

Node	Region	BA/nucleus	Degree	MNI coord [mm]			Region
				*x*	*y*	*z*	
57	Inferior temporal	21	4	47	4	−40	DMN
44	Precuneus	7	4	8	−57	62	DMN
191	Middle temporal	22	3	−59	−30	4	DMN
187	Temporal pole	21	3	−50	11	−31	FPN
148	Sup medial frontal	8	3	−11	34	51	DMN

Networks: DAN, dorsal attention; DMN, default-mode; VN, visual; FPN, frontoparietal. Node numbers correspond to the Shen atlas.

Next, we tested the prediction power of these overlapping features in the Training and Test subsamples. Specifically, we computed network strength from positive and negative edges of the overlapping networks, which we refer to as ‘*motion-sensitive networks*’ and found that these features predicted head motion in all fMRI sessions with similar accuracy in the Training and Test subsamples, both for *d* and Δ*d* (Figure 8C, left and middle panels).

Further support for the involvement of these motion-sensitive networks in head motion prediction emerged from an independent validation study in 1,422 healthy young adults who underwent resting-state fMRI with standard spatiotemporal resolution (3 mm-isotropic; 3 s TR) under the GSP study (Holmes et al., 2015). Specifically, we found that motion-sensitive network strengths, computed using the positive and negative edges defined in the HCP absolute motion-sensitive network and the corresponding parameters of the bilinear model (Figure 8A), predicted *d* (0.13 mm ± 0.08 mm; mean ± sd) in two age- and gender-matched samples of 711 individuals: GSP1 (21.5 ± 2.9 years; 394 females) and GSP2 (21.5 ± 2.9 years; 406 females), with similar accuracy to that in the HCP subsamples (R ~ 0.3; Figure 8C). However, the predictability of Δ*d* (0.04 mm ± 0.02 mm/TR; mean ± sd) in the GSP datasets, based on the Δ*d*-motion-sensitive network and the parameters of the bilinear model (Figure 8B), was not consistent across GSP1 and GSP2 (Figure 8C). In simulated data, the parameters of the bilinear model and positive and negative edges of the *d*- and Δ*d*-motion-sensitive networks (Figures 7B, 8A) predicted Δ*d* in GSP1 and *d* in GSP2, with significantly lower accuracy than in real data (Figure 8C).

## Discussion

Here we identify two motion-sensitive networks that predicted individual differences in head motion across six different task-fMRI and two rest-fMRI sessions. Reproducible predictions emerged from a Training sample of 207 individuals, using internal validation, and from an independent sample of 207 novel individuals, using twofold cross-validation. Head motion prediction was robust to changes in motion metric (*d* or Δ*d*), task-rest condition, brain parcellation, and model, demonstrating that results were stable and reproducible. We further validated our head motion prediction model in two independent datasets of 711 individuals, but similar validations failed in simulated datasets without neurobiological contributions. The predictability of head motion despite the relatively small frame-to-frame translations in this work (Δ*d* ~ 0.04 mm), compared to the stringent 0.2 mm micro motion threshold (Van Dijk et al., 2012), suggests that even extremely low amounts of head motion can influence functional connectivity.

The predictability of *d* is both surprising and interesting because “absolute motion” is a summary measure of slow motion rather than one of velocity. Here we show for the first time that the cerebellum (Crus II) reliably contributed to the prediction of *d*, whereas lateral DMN components (temporal cortex) contributed to the prediction of Δ*d*. The specificity of the cerebellum to *d*-prediction accuracy suggests that Crus II is particularly sensitive to slow head motion. The specificity of the lateral temporal DMN areas to Δ*d*-prediction accuracy suggests that this DMN subsystem is particularly sensitive to rapid head motion. However, the highly reproducible predictions of motion from within- and between-network edges (Figure 6) is consistent with the notion that all the networks are associated with head movement (Satterthwaite et al., 2013a), and not only the Crus II and temporal DMN regions.

A frequent approach to control for motion in functional connectivity studies is to exclude data with large Δ*d* (Power et al., 2012; Satterthwaite et al., 2012; Van Dijk et al., 2012) while little attention is given to *d*. However, our data shows that excluding data based on Δ*d* may not be sufficient to warrant the absence of motion effects on functional connectivity. Indeed, we show that motion prediction was higher for *d* than Δ*d*. Furthermore, functional connectivity data predicted both *d* and Δ*d* despite the use of current methods to attenuate the influence of head motion on fMRI. Prior studies showed that scrubbing with FD > 0.2 mm attenuated negative (but not positive) correlations between head motion (i.e., the average residual FD) and fMRI signals suggesting that negative relationships are likely to originate from motion artifacts (Yan et al., 2013). However, prediction accuracy from datasets with low motion (Δ*d* ~ 0.04 mm) did not differ when computed from positive or negative edges or when using stringent (FD > 0.2 mm) or lenient (FD > 0.5 mm) scrubbing thresholds.

Task-based fMRI studies, which frequently restrict head movement to minimize task-correlated motion artifacts, have demonstrated that older adults and patient populations move more during scanning than healthy controls (Seto et al., 2001; Yuan et al., 2009; Haller et al., 2014). Moreover, some have suggested that in-scanner head motion could be heritable (Engelhardt et al., 2017). Head motion is particularly problematic for resting state fMRI studies in pediatric populations, where an inverse relationship exists between head motion and age (Frew et al., 2022). Note that children have greater difficulty in staying still during scanning than adults, due in part to the incomplete maturation of prefrontal cortical regions necessary for self-regulation, and also their propensity to boredom and anxiety (Morel et al., 2019). However, anxiety concerns are also relevant to adults, many of whom report some level of anxiety when undergoing MRI scans (Dziuda et al., 2019).

Pathological conditions or demographic parameters such as age can influence in-scanner head motion (Saccà et al., 2021). Thus, our findings on young healthy subjects could differ from those in older or pathological populations. Future studies are needed to investigate the extent to which this could be disentangled into contributions from the different components of the displacement (i.e., translational, rotational) and the effect of respiratory artifacts (Fair et al., 2020). It is challenging to explore all possible alterations in image preprocessing choices that might potentially interact with head motion. The use of different MRI acquisition protocols and preprocessing pipelines could reduce the reproducibility across HCP and GSP datasets. Nevertheless, the use of a different image acquisition protocol and preprocessing pipeline in the GSP and HCP datasets, allowed us to generalize our findings on motion prediction to different image acquisition protocols and preprocessing pipelines.

Since deleterious effects of head motion on fMRI data are well-documented (Power et al., 2012; Satterthwaite et al., 2012; Van Dijk et al., 2012) and considering that true brain-behavior associations have small effects (Marek et al., 2022) the predictions of head motion in this work likely reflect correlations of time-varying artifacts among brain regions that are highly sensitive to motion. However, the poor prediction accuracy obtained with simulated fMRI data reflecting rigid-body motion contrasts with the successful validation of the prediction model in real data (Figure 8C), does not allow us to rule out the potential contributions of neurobiological origins in the prediction of *d*.

Together, our findings show that functional connectivity is a reproducible predictor of head motion and identify cerebellar and DMN subsystems that are highly sensitive to absolute and relative micromotion.

## Data availability statement

The datasets presented in this study can be found in online repositories. The names of the repository/repositories and accession number(s) can be found at: Brain Genomics Superstruct Project (GSP) https://www.neuroinfo.org/gsp and Human Connectome Project (HCP) http://www.humanconnectome.org/.

## Ethics statement

The studies involving human participants were reviewed and approved by IRB at Washington University and Partners Health Care Institutional Review Board and the Harvard University Committee. The patients/participants provided their written informed consent to participate in this study.

## Author contributions

DT and NV: study conception and design, interpretation of results, and draft manuscript preparation. DT: data analyses. All authors contributed to the article and approved the submitted version.

## Funding

This work was accomplished with support from the National Institutes of Alcohol Abuse and Alcoholism (ZIAAA000550).

## Conflict of interest

The authors declare that the research was conducted in the absence of any commercial or financial relationships that could be construed as a potential conflict of interest.

## Publisher’s note

All claims expressed in this article are solely those of the authors and do not necessarily represent those of their affiliated organizations, or those of the publisher, the editors and the reviewers. Any product that may be evaluated in this article, or claim that may be made by its manufacturer, is not guaranteed or endorsed by the publisher.

## Footnotes

^1^ http://www.humanconnectome.org/

^2^ https://www.neuroinfo.org/gsp

^3^ http://surfer.nmr.mgh.harvard.edu

^4^ http://www.fmrib.ox.ac.uk/fsl
